# Erythropoietic effects of vadadustat in patients with anemia associated with chronic kidney disease

**DOI:** 10.1002/ajh.26644

**Published:** 2022-07-15

**Authors:** Mark J. Koury, Rajiv Agarwal, Glenn M. Chertow, Kai‐Uwe Eckardt, Steven Fishbane, Tomas Ganz, Volker H. Haase, Mark R. Hanudel, Patrick S. Parfrey, Pablo E. Pergola, Prabir Roy‐Chaudhury, James A. Tumlin, Robert Anders, Youssef M. K. Farag, Wenli Luo, Todd Minga, Christine Solinsky, Dennis L. Vargo, Wolfgang C. Winkelmayer

**Affiliations:** ^1^ Division of Hematology/Oncology, Department of Medicine Vanderbilt University Medical Center Nashville Tennessee USA; ^2^ Department of Medicine, Division of Nephrology Indiana University School of Medicine Indianapolis Indiana USA; ^3^ Stanford University School of Medicine Palo Alto California USA; ^4^ Department of Nephrology and Medical Intensive Care Charité – Universitätsmedizin Berlin Berlin Germany; ^5^ Division of Nephrology, Department of Medicine Hofstra Northwell School of Medicine Great Neck New York USA; ^6^ Department of Medicine and Pathology, David Geffen School of Medicine University of California Los Angeles California USA; ^7^ Department of Medicine Vanderbilt University Medical Center Nashville Tennessee USA; ^8^ Department of Medical Cell Biology Uppsala University Uppsala Sweden; ^9^ Department of Pediatrics, Division of Pediatric Nephrology, David Geffen School of Medicine University of California Los Angeles California USA; ^10^ Department of Medicine Memorial University St John's Newfoundland and Labrador Canada; ^11^ Renal Associates PA San Antonio Texas USA; ^12^ W.G. (Bill) Hefner VA Medical Center Salisbury North Carolina USA; ^13^ Emory University School of Medicine Atlanta Georgia USA; ^14^ Akebia Therapeutics, Inc. Cambridge Massachusetts USA; ^15^ Section of Nephrology Baylor College of Medicine Houston Texas USA

## Abstract

Patients with chronic kidney disease (CKD) develop anemia largely because of inappropriately low erythropoietin (EPO) production and insufficient iron available to erythroid precursors. In four phase 3, randomized, open‐label, clinical trials in dialysis‐dependent and non–dialysis‐dependent patients with CKD and anemia, the hypoxia‐inducible factor prolyl hydroxylase inhibitor, vadadustat, was noninferior to the erythropoiesis‐stimulating agent, darbepoetin alfa, in increasing and maintaining target hemoglobin concentrations. In these trials, vadadustat increased the concentrations of serum EPO, the numbers of circulating erythrocytes, and the numbers of circulating reticulocytes. Achieved hemoglobin concentrations were similar in patients treated with either vadadustat or darbepoetin alfa, but compared with patients receiving darbepoetin alfa, those receiving vadadustat had erythrocytes with increased mean corpuscular volume and mean corpuscular hemoglobin, while the red cell distribution width was decreased. Increased serum transferrin concentrations, as measured by total iron‐binding capacity, combined with stable serum iron concentrations, resulted in decreased transferrin saturation in patients randomized to vadadustat compared with patients randomized to darbepoetin alfa. The decreases in transferrin saturation were associated with relatively greater declines in serum hepcidin and ferritin in patients receiving vadadustat compared with those receiving darbepoetin alfa. These results for serum transferrin saturation, hepcidin, ferritin, and erythrocyte indices were consistent with improved iron availability in the patients receiving vadadustat. Thus, overall, vadadustat had beneficial effects on three aspects of erythropoiesis in patients with anemia associated with CKD: increased endogenous EPO production, improved iron availability to erythroid cells, and increased reticulocytes in the circulation.

## 1 INTRODUCTION

Chronic kidney disease (CKD) is associated with anemia that worsens as kidney function declines.^1^ In a representative sample of the U.S. population with stages 4 and 5 CKD, indicated by an estimated glomerular filtration rate (eGFR) of <30 mL/min/1.73 m^2^, approximately one‐half had anemia.^2^ The primary cause of anemia in CKD is underproduction of red blood cells (RBCs).^3^ Erythropoietin (EPO), which is mainly produced by a subset of renal cortical interstitial fibroblasts in response to local tissue hypoxia,^4^, ^5^ prevents apoptosis of marrow erythroid progenitors.^6^, ^7^ Decreased EPO production results from inflammation in the kidneys^8^, ^9^ when renal EPO‐producing fibroblasts are transformed into myofibroblasts that do not produce EPO.^8^

Inflammation in CKD also directly suppresses erythroid cell development and indirectly suppresses the iron available to erythroblasts via hepcidin induction.^10^, ^11^, ^12^ Hepcidin, a liver peptide induced by inflammation and/or increased iron, binds and downregulates cell surface expression of ferroportin, the transmembrane iron exporter.^13^ Increased hepcidin in CKD restricts iron export from storage cells, macrophages, and hepatocytes, thereby decreasing iron availability to erythroblasts.^14^, ^15^ Macrophages remove senescent erythrocytes from the blood and export iron recycled from degraded hemoglobin to marrow erythropoietic cells.^16^, ^17^ In addition, erythrocyte lifespan in CKD is decreased, likely by inflammation‐induced macrophage activation and uremic toxin‐induced damage to erythrocytes.^18^ Chronic blood loss from retention in the hemodialysis apparatus further limits erythrocyte lifespan and iron availability.^11^ Thus, anemia in CKD results from decreased EPO production, suppression of erythroid progenitors, restricted iron availability, and shortened erythrocyte lifespan.

Recombinant human EPO (rhEPO) and its derivatives with prolonged half‐lives, darbepoetin alfa (hereafter termed “darbepoetin”) and polyethylene‐glycolated rhEPO, are collectively termed erythropoiesis‐stimulating agents (ESAs), and are standard treatments for anemic patients with CKD that reduce the need for blood transfusions.^19^ However, ESAs are associated with adverse cardiovascular events when hemoglobin concentrations in the normal or slightly subnormal ranges are targeted.^20^, ^21^, ^22^ Among potential therapeutic agents for anemia in patients with CKD are those that induce endogenous EPO production, thereby avoiding high transient plasma concentrations of ESAs that may occur with intravenous administration.

EPO production is controlled by hypoxia‐inducible factor (HIF).^23^, ^24^ HIF, a multicomponent transcription factor, is mainly regulated by the degradation rate of one of its components, HIFα,^25^ which is targeted for polyubiquitination and proteasomal degradation by prolyl hydroxylation.^26^, ^27^, ^28^ Three specific HIF prolyl hydroxylases (HIF prolyl‐4‐hydroxylase domain [HIF‐PHD] enzymes) use oxygen, 2‐oxoglutarate, and one of three HIFα proteins (HIF‐1α, HIF‐2α, or HIF‐3α) as substrates.^29^, ^30^, ^31^ Under normoxic conditions, HIFα is rapidly hydroxylated and promptly degraded so that it does not accumulate and form active transcription factor complexes. With hypoxia, the HIFα prolines are not hydroxylated, and HIFα is incorporated into active HIF transcription factor complexes that bind to hypoxia‐responsive elements in many target genes, including the gene encoding EPO, thereby inducing transcription of those genes.^32^

Vadadustat, an investigational oral HIF‐PHD inhibitor that stabilizes intracellular HIFα, increases serum EPO concentrations over baseline in healthy individuals and in patients with CKD.^33^, ^34^ In four phase 3 clinical trials, vadadustat was compared with darbepoetin for efficacy and safety in the treatment of anemia associated with non‐dialysis‐dependent CKD (NDD‐CKD; i.e., the PRO_2_TECT program, two trials) and dialysis‐dependent CKD (DD‐CKD; i.e., the INNO_2_VATE program, two trials). The INNO_2_VATE and PRO_2_TECT trials both met the prespecified noninferiority criteria (−0.75 g/dL) for the primary efficacy endpoint of mean change in hemoglobin from baseline to Weeks 24 to 36. The INNO_2_VATE trial also met the prespecified noninferiority criteria (1.25) for the primary safety endpoint of time to first major adverse cardiovascular event (MACE; a composite of death from any cause, nonfatal myocardial infarction, or a nonfatal stroke); however, the PRO_2_TECT trials did not meet the prespecified margin for noninferiority.^35^, ^36^ Time to CKD progression in the PRO_2_TECT trials (provision of maintenance dialysis, receipt of kidney transplant, or decrease in eGFR ≤15 mL/min/1.73 m^2^ or a decrease of 40% or more in the eGFR with the two eGFR criteria confirmed after ≥4 weeks), was similar in the vadadustat‐ and darbepoetin‐treatment groups.^35^ Here, we report the erythropoietic effects of vadadustat in these trials and discuss their clinical relevance.

## 2 METHODS

### 2.1 Trial designs and oversight

We conducted four global, open‐label, sponsor‐blinded, active‐controlled, noninferiority trials in patients with CKD. The two PRO_2_TECT trials included ESA‐untreated patients with NDD‐CKD (ClinicalTrials.gov identifier: NCT02648347) or ESA‐treated patients with NDD‐CKD (ClinicalTrials.gov identifier: NCT02680574); the two INNO_2_VATE trials included ESA‐treated patients with incident DD‐CKD (dialysis initiated within 16 weeks before screening; ClinicalTrials.gov identifier: NCT02865850) and prevalent DD‐CKD (dialysis received for at least 12 weeks before screening; ClinicalTrials.gov identifier: NCT02892149).^35^, ^36^ Flow diagrams showing randomization to either vadadustat or darbepoetin and subsequent trial events are shown for the PRO_2_TECT trials in Figure S1 and for the INNO_2_VATE trials in Figure S2.

### 2.2 Patients and assessments

In both PRO_2_TECT trials, eligible patients were at least 18 years of age and had NDD‐CKD (eGFR ≤60 mL/min/1.73 m^2^). In the trial involving ESA‐untreated patients, patients were required to have a hemoglobin concentration <10 g/dL and were excluded if they had received any ESA within 8 weeks before randomization. In the trial involving ESA‐treated patients, patients had to be actively receiving maintenance ESA therapy, with at least one dose received within 6 weeks before or during screening and were required to have a hemoglobin concentration of 8–11 g/dL (in the United States) or 9–12 g/dL (in other countries). In both INNO_2_VATE trials, eligible patients were at least 18 years of age, had CKD and were undergoing dialysis, had a serum ferritin concentration ≥100 ng/mL and a transferrin saturation (TSAT) ≥20%, and had not received an RBC transfusion within the previous 8 weeks. In addition, patients had a hemoglobin concentration between 8 and 11 g/dL (incident DD‐CKD trial) or a hemoglobin concentration between 8 and 11 g/dL (in the United States) or between 9 and 12 g/dL (in other countries) (prevalent DD‐CKD trial). In all trials, oral or intravenous iron administration was allowed at the discretion of local investigators to maintain serum ferritin ≥100 ng/mL or TSAT ≥20%.

The PRO_2_TECT and INNO_2_VATE trials had prespecified primary (Weeks 24–36) and secondary (Weeks 40–52) evaluation periods for efficacy, as measured by change or maintenance of hemoglobin concentrations (target hemoglobin concentrations of 10–11 g/dL in the United States and 10–12 g/dL in other countries). The prespecified noninferiority margins were selected in consultation with regulatory agencies as the lower bound of the 95% confidence interval of −0.75 g/dL for the primary efficacy end point. The primary safety outcome was time to first MACE with a prespecified noninferiority margin of 1.25.

In addition to baseline values, erythrocyte numbers (RBCs) and erythrocyte indices were measured every 2 weeks through Week 12, then every fourth week. Serum ferritin, iron, TSAT, and total iron‐binding capacity (TIBC), were measured every 4 weeks through Week 12, and every 8 weeks through Week 52. Serum bilirubin and lactate dehydrogenase were measured every 4 weeks through Week 28, and every 8 weeks through Week 52. Reticulocyte numbers and serum hepcidin (measured by enzyme‐linked immunoassay [ELISA]; DRG International Inc.) were measured at Weeks 4, 12, 28, and 52; serum C‐reactive protein (CRP; measured by an immunoturbidimetric assay; Roche Diagnostics) was measured at Weeks 28 and 52. Endogenous serum EPO was measured at Weeks 4, 12, 28, and 52 in patients randomized to receive vadadustat using an ELISA (Quantikine IVD; R&D Systems). Endogenous EPO could not be measured in patients randomized to receive darbepoetin because, as a derivative of EPO, darbepoetin is immunologically related to EPO and darbepoetin's erythropoietic activity suppresses endogenous EPO production. However, serum EPO concentrations were also determined in darbepoetin‐treated patients, while recognizing that their values represented combined endogenous EPO and exogenous darbepoetin.

### 2.3 Data analysis and statistical methods

We analyzed erythropoietic effects of vadadustat for the safety population (all randomized patients who received at least one dose of study drug). Blood samples for laboratory assays were sent to the trials' central laboratory for analysis. Local investigators were responsible for reviewing laboratory results for clinical significance and adjusting the dose according to the protocol algorithm. Results for RBCs, reticulocytes, mean corpuscular volume (MCV), mean corpuscular hemoglobin (MCH), red cell distribution width (RDW; a measure of anisocytosis), and serum EPO, TIBC, iron, TSAT, hepcidin, and ferritin were compared with respect to mean change from baseline in the primary and secondary evaluation periods. Within treatment group analyses were based on paired *t* tests. The analyses between groups were based on analyses of covariance with the baseline values of laboratory tests as covariates, and central laboratory baseline hemoglobin group, geographic region, New York Heart Association congestive heart failure class, and treatment group as fixed effects. We considered two‐tailed *p* < .05 as statistically significant without adjustment for multiple comparisons. All analyses were conducted using SAS statistical software version 9.4 (SAS Institute Inc).

## 3 RESULTS

In the PRO_2_TECT trials, 1751 ESA‐untreated patients with NDD‐CKD and 1725 ESA‐treated patients with NDD‐CKD (3476 patients in total) were randomly assigned in a 1:1 ratio to receive vadadustat or darbepoetin. In the INNO_2_VATE trials, 369 patients in the incident DD‐CKD trial and 3554 patients in the prevalent DD‐CKD trial (3923 patients in total) were randomly assigned in a 1:1 ratio to receive vadadustat or darbepoetin. Baseline characteristics were generally similar among patients randomized to vadadustat or darbepoetin within all trials (Table S1).

### 3.1 EPO concentrations, hemoglobin concentrations, and RBCs

Mean serum EPO concentrations were measured in patients treated with vadadustat in the NDD‐CKD and DD‐CKD trial populations to determine the effects of vadadustat on endogenous EPO production. Patients in the ESA‐untreated NDD‐CKD trial were the only ones with an ESA‐naïve baseline, and therefore, the only group in which an induction in endogenous EPO could be reliably determined. Mean serum EPO concentrations were statistically significantly increased over baseline at each time point (4, 12, 28, and 52 weeks) in both the vadadustat‐treatment and darbepoetin‐treatment groups while remaining in the 16–20 mIU/mL range (Tables 1 and S2). These increases were due to endogenous EPO production in the vadadustat‐treated group (Table 1) and combined exogenous darbepoetin and endogenous EPO in the darbepoetin‐treated group (Table S2).

**TABLE 1:** Erythropoietin levels in vadadustat‐treated patients at baseline and Weeks 4, 12, 28, and 52 in the PRO_2_TECT and INNO_2_VATE programs

Erythropoietin (mIU/mL)	Baseline	Week 4	Week 12	Week 28	Week 52
ESA‐untreated NDD‐CKD trial					
Observed, mean (SD)	15.2 (14.0)	16.2 (15.2)	17.3 (23.3)	19.6 (47.2)	20.1 (37.2)
Change from baseline, mean (SD)	NA	1.3 (10.7)	2.3 (22.6)	5.0 (47.4)	5.2 (38.1)
Change from baseline, 95% CI	NA	(0.5, 2.0)	(0.7, 4.0)	(1.4, 8.6)	(1.8, 8.6)
*p* value		.0008	.005	.007	.0028
ESA‐treated NDD‐CKD trial					
Observed, mean (SD)	15.4 (14.9)	14.7 (14.6)	16.7 (46.7)	17.4 (38.7)	21.1 (46.0)
Change from baseline, mean (SD)	NA	−0.7 (15.3)	1.4 (47.2)	2.1 (40.3)	5.7 (47.0)
Change from baseline, 95% CI	NA	(−1.8, 0.3)	(−1.9, 4.8)	(−0.8, 5.1)	(1.6, 9.8)
*p* value		.1759	.405	.1578	.0064
Incident DD‐CKD trial					
Observed, mean (SD)	17.8 (29.3)	20.3 (41.6)	22.4 (49.7)	19.9 (42.8)	17.5 (19.9)
Change from baseline, mean (SD)	NA	2.5 (46.3)	4.5 (57.1)	1.9 (52.6)	−3.5 (40.8)
Change from baseline, 95% CI	NA	(−4.5, 9.6)	(−4.4, 13.4)	(−6.6, 10.4)	(−12.0, 5.0)
*p* value		.4845	.3168	.655	.418
Prevalent DD‐CKD trial					
Observed, mean (SD)	26.3 (76.0)	20.4 (39.3)	27.0 (84.3)	22.7 (51.5)	32.1 (101.4)
Change from baseline, mean (SD)	NA	−6.5 (84.1)	0.3 (110.3)	−2.2 (83.3)	6.3 (116.5)
Change from baseline, 95% CI	NA	(−10.5, −2.4)	(−5.2, 5.7)	(−6.5, 2.1)	(−0.4, 13.0)
*p* value		.0019	.9273	.3096	.0633

Abbreviations: CKD, chronic kidney disease; DD, dialysis dependent; ESA, erythropoiesis‐stimulating agent; NA, not applicable; NDD, non‐dialysis dependent.

In both treatment groups, mean serum EPO levels in the ESA‐treated NDD‐CKD trial were in ranges similar to those in the ESA‐untreated trial. In the incident and prevalent DD‐CKD trials, both vadadustat‐treated and darbepoetin‐treated patients maintained their serum EPO levels that were slightly increased at baseline compared with those of patients in the NDD‐CKD trials (20–32 mIU/mL) (Tables 1 and S2).

**FIGURE 1:**
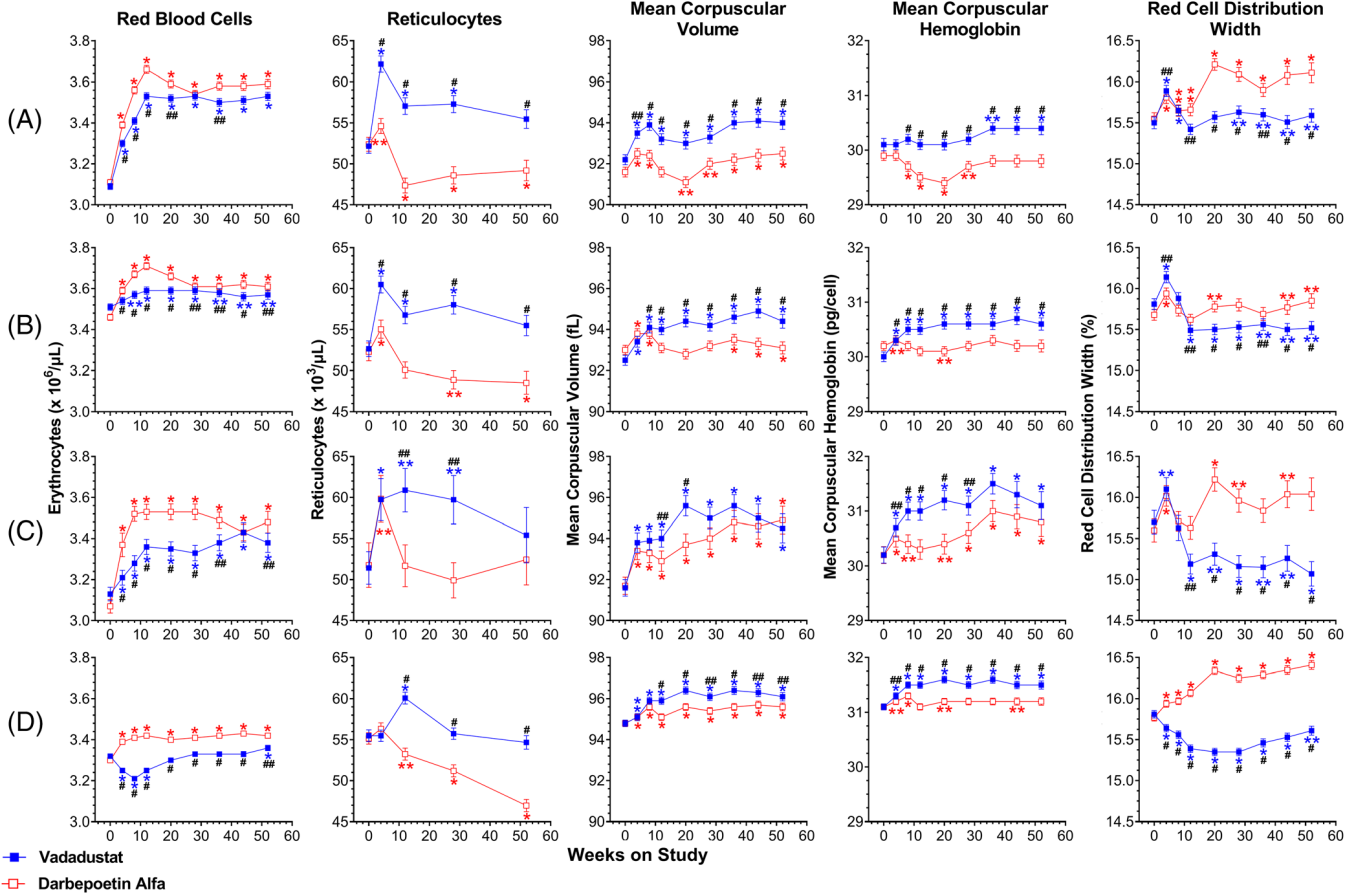
Red blood cells, reticulocytes, mean corpuscular volume, mean corpuscular hemoglobin, and red cell distribution width of patients in phase 3 trials evaluating vadadustat versus darbepoetin alfa for treatment of anemia in chronic kidney disease. (A) ESA‐untreated NDD‐CKD trial; (B) ESA‐treated NDD‐CKD trial; (C) incident DD‐CKD trial; (D) prevalent DD‐CKD trial. DD‐CKD, dialysis‐dependent chronic kidney disease; NDD‐CKD, non‐dialysis‐dependent chronic kidney disease; ESA, erythropoiesis‐stimulating agent. Data points for vadadustat‐treated patients (solid blue squares) and darbepoetin‐treated patients (open red squares) represent means; error bars represent standard error of the mean. Within‐group difference from baseline: *p ≤ .001, **p ≤ .05. Between‐group difference: ^#^ *p* ≤ .001, ^##^ *p* ≤ .05

In all four trials, vadadustat was noninferior to darbepoetin in achieving and maintaining target hemoglobin concentrations.^35^, ^36^ In the trial in ESA‐untreated NDD‐CKD patients, hemoglobin increased from 9.1 g/dL at baseline to 10.6 g/dL at Week 52 in vadadustat‐ and darbepoetin‐treatment groups (Table S3). RBCs increased from 3.1 × 10^6^/μL in both groups at baseline to 3.5 and 3.6 × 10^6^/μL at Week 52 in the vadadustat‐ and darbepoetin‐treatment groups, respectively (Table S3 and Figure 1). In ESA‐treated NDD‐CKD patients, hemoglobin increased from 10.4 g/dL at baseline to 10.8 g/dL at Week 52 in both vadadustat‐ and darbepoetin‐treatment groups (Table S3). RBCs were 3.5 × 10^6^/μL at baseline and 3.6 × 10^6^/μL at Week 52 in vadadustat‐ and darbepoetin‐treatment groups (Table S3 and Figure 1).

In incident DD‐CKD patients, hemoglobin increased from 9.4 and 9.2 g/dL at baseline to 10.4 and 10.6 g/dL at Week 52 in the vadadustat‐ and darbepoetin‐treatment groups, respectively (Table S3). RBCs increased from 3.1 × 10^6^/μL at baseline in both groups to 3.4 and 3.5 × 10^6^/μL at Week 52 in the vadadustat‐ and darbepoetin‐treatment groups, respectively (Table S3 and Figure 1). In the prevalent DD‐CKD group of patients already being treated with ESAs, hemoglobin increased from 10.3 and 10.2 g/dL at baseline to 10.5 and 10.6 g/dL at Week 52 in the vadadustat‐ and darbepoetin‐treatment groups, respectively (Table S3).

In both trials in incident and prevalent DD‐CKD patients, the respective achievement and maintenance of hemoglobin were associated with parallel increases in RBCs/μL (Table S3 and Figure 1). RBCs increased from 3.3 × 10^6^/μL at baseline to 3.4 × 10^6^/μL at Week 52 in both vadadustat‐ and darbepoetin‐treatment groups (Table S3 and Figure 1).

### 3.2 Effects on reticulocyte numbers

Absolute reticulocytes were statistically significantly increased by approximately 10%–20% in the vadadustat‐treatment groups compared with stable absolute reticulocyte numbers in the darbepoetin‐treatment groups at Weeks 4–52 in ESA‐untreated NDD‐CKD patients, Weeks 4–52 in the trial in ESA‐treated NDD‐CKD patients, Weeks 12 and 28 in incident DD‐CKD patients, and Weeks 12–52 in prevalent DD‐CKD patients (Table S3 and Figure 1). Serum total bilirubin and lactate dehydrogenase concentrations were similar in the vadadustat and darbepoetin arms throughout the trials, indicating no increase in rate of hemolysis in the vadadustat‐treatment groups that would account for the differences in reticulocytes (Figure S3). Furthermore, adverse event reporting provided no evidence for increased bleeding in the vadadustat‐ versus the darbepoetin‐treatment group in each trial.

### 3.3 Effects on erythrocyte indices

MCV and MCH values were within the population reference range (MCV 79–98 fL; MCH 26–34 pg/cell), although mean MCV and mean MCH were consistently higher in patients randomized to vadadustat compared with those randomized to darbepoetin at each time point in all four trials (Table S3 and Figure 1). The increased MCV with vadadustat treatment was uniformly seen across the four trials, as demonstrated by decreased RDW with vadadustat treatment compared with darbepoetin treatment (Table S3 and Figure 1). Across each trial, the MCV of non‐reticulocytes in the RBC population at Weeks 28 and 52 showed the same MCV relationships as shown for the total RBC population (Table S4).

### 3.4 Effects on TIBC, serum iron, and TSAT

**FIGURE 2:**
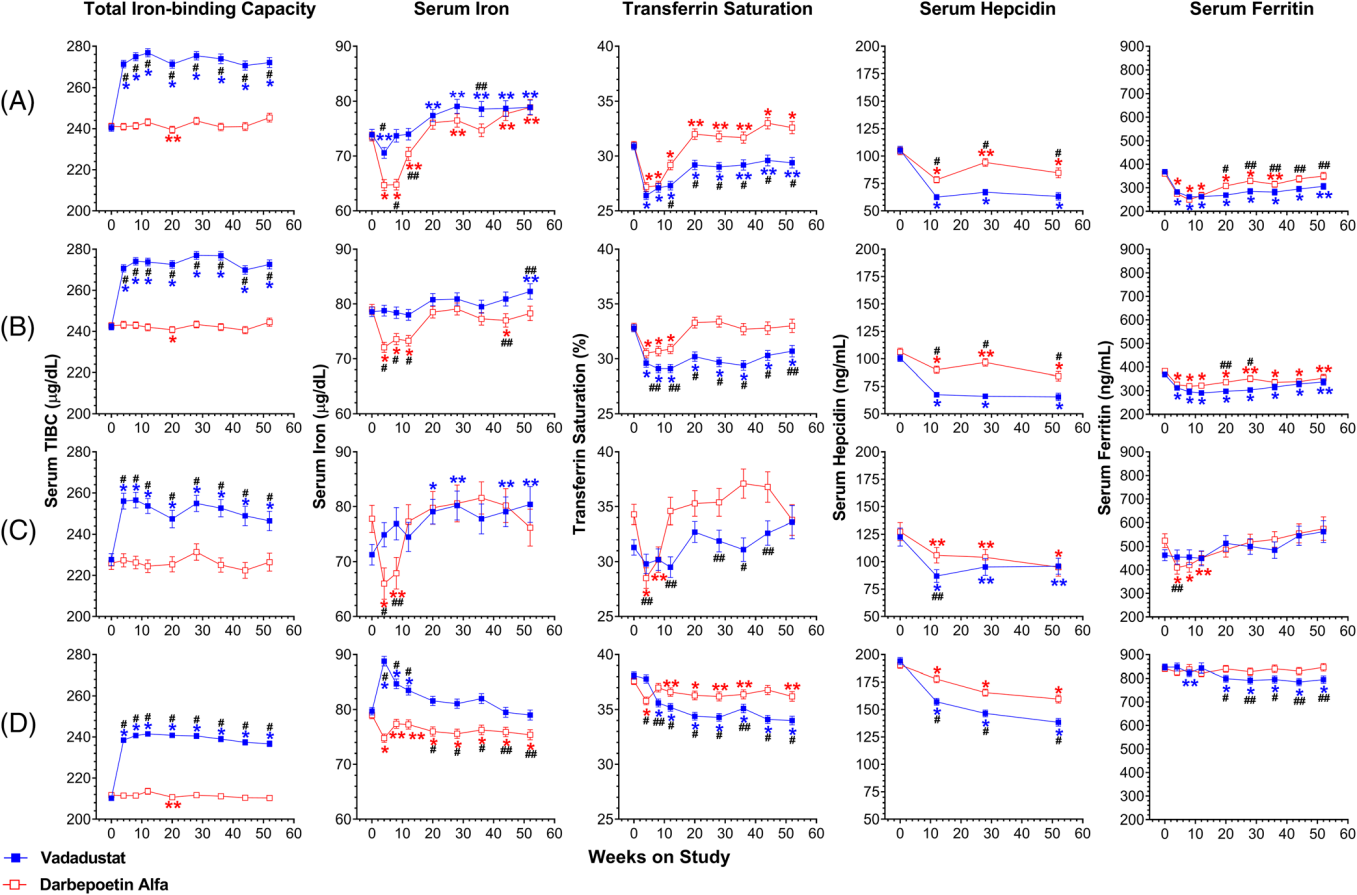
Serum total iron binding capacity, serum iron, transferrin saturation, serum hepcidin, and serum ferritin of patients in phase 3 trials evaluating vadadustat versus darbepoetin alfa for treatment of anemia in chronic kidney disease. (A) ESA‐untreated NDD‐CKD trial; (B) ESA‐treated NDD‐CKD trial; (C) incident DD‐CKD trial; (D) prevalent DD‐CKD trial. DD‐CKD, dialysis‐dependent chronic kidney disease; NDD‐CKD, non‐dialysis‐dependent chronic kidney disease; ESA, erythropoiesis‐stimulating agent. Data points for vadadustat‐treated patients (solid blue squares) and darbepoetin‐treated patients (open red squares) represent means; error bars represent standard error of the mean. Within‐group difference from baseline: *p ≤ .001, **p ≤ .05. Between‐group difference: ^#^ *p* ≤ .001, ^##^ *p* ≤ .05

In all four trials, serum transferrin concentrations as measured by mean TIBC were significantly increased over baseline at all times in patients randomized to vadadustat relative to those in patients randomized to darbepoetin (Figure 2 and Table S5). Mean baseline TIBC concentrations in all groups were below the lower limits of the population reference range (250–425 μg/dL),^37^ and patients with NDD‐CKD randomized to vadadustat had increases in TIBC concentrations to within the (lower) population reference range. In patients randomized to vadadustat, mean serum iron concentrations were increased from baseline in the primary (Weeks 24–36) and secondary (Weeks 40–52) efficacy evaluation periods, except in the prevalent DD‐CKD group where it remained unchanged (Figure 2 and Table S5). Over the course of both PRO_2_TECT trials and the trial in prevalent DD‐CKD, serum iron was significantly higher in the vadadustat‐treated group compared with the darbepoetin‐treated groups (Figure 2 and Table S5). Before the primary evaluation period, serum iron was significantly higher in the vadadustat‐treated group compared with the darbepoetin‐treated group in the trial in incident DD‐CKD patients (Figure 2 and Table S5).

Across all four trials, the generalized increases in TIBC in vadadustat‐treated groups resulted in a pattern of decreased mean TSAT in the early weeks of each trial followed by recovery of mean TSAT only in the incident DD‐CKD trial (Figure 2 and Table S5). In darbepoetin‐treated groups, across all four trials, mean TSAT transiently decreased followed by a recovery of mean TSAT toward baseline, although this recovery was more limited in the prevalent DD‐CKD trial (Figure 2 and Table S5).

### 3.5 Effect on serum hepcidin and ferritin

Mean serum hepcidin concentrations decreased in both treatment groups in each trial, with more prominent decreases in patients randomized to vadadustat than in those randomized to darbepoetin (Figure 2 and Table S5). Baseline mean serum concentrations of ferritin, the major intracellular iron storage protein, were at or above the upper limits of the population reference range (10–380 ng/mL) in all trials (Figure 2 and Table S5), but serum ferritin decreased at Weeks 4 and 12, when erythropoiesis was induced in the NDD‐CKD trials and the incident DD‐CKD trial (Figure 2 and Table S5). By the primary evaluation period at Weeks 24–36 and secondary evaluation period at Weeks 40–52, mean serum ferritin had increased over the earlier nadirs at Weeks 4 and 12 in both NDD‐CKD trials to slightly less than baseline concentrations (Figure 2 and Table S5). In the incident DD‐CKD trial, the slight decreases in mean serum ferritin of the darbepoetin‐treated group and no change in the vadadustat‐treated group at Weeks 4 and 12 were followed by increases at Weeks 28 and 52, so that both arms had concentrations at or above baseline (Figure 2 and Table S5). The prevalent DD‐CKD group, which had extremely high baseline mean serum ferritin, showed little change throughout the trial in patients receiving darbepoetin, and a sustained decrease in mean serum ferritin beginning at Week 20 in the group receiving vadadustat (Figure 2). As inflammation induces hepcidin, CRP was measured, and the mean CRP was slightly to moderately elevated in the 7–11 mg/L range at baseline (CRP population reference range: 0.0–4.9 mg/L)^38^ in all groups and remained in this range at all time points (Figure S3).

## 4 DISCUSSION

**FIGURE 3:**
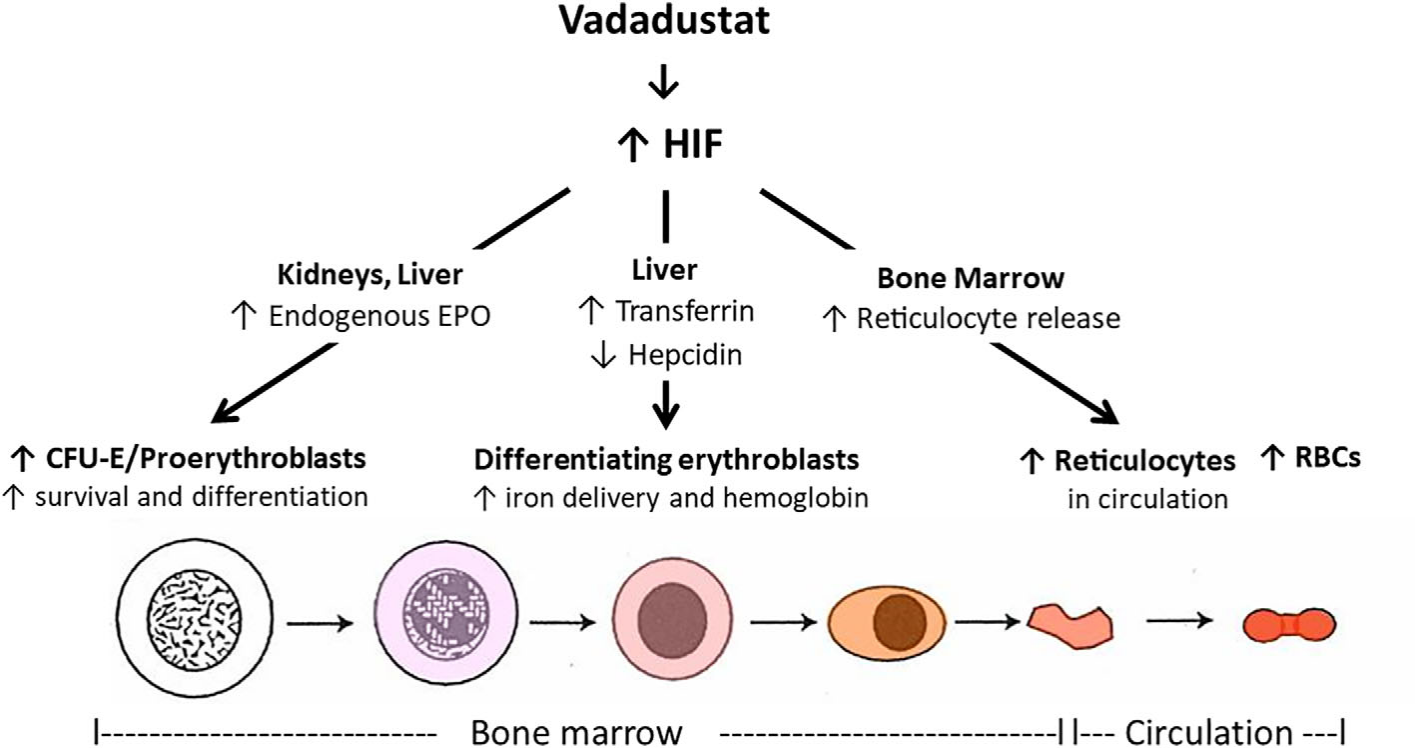
Vadadustat effects on erythropoiesis. Vadadustat pharmacologically increases hypoxia‐inducible factor (HIF) and transcription of HIF target genes. In the kidneys and liver, vadadustat improves production of endogenous erythropoietin (EPO), which is insufficient in chronic kidney disease. EPO increases survival and differentiation of marrow erythroid progenitors (colony‐forming units erythroid/proerythroblasts), thereby expanding red blood cell (RBC) production. In the liver, vadadustat increases production of transferrin, the plasma iron carrier, and decreases production of hepcidin, the negative regulator of ferroportin, which exports iron from duodenal enterocytes (where iron is absorbed) and hepatocytes and macrophages (where iron is stored). Increased iron availability and its delivery by transferrin to marrow erythroblasts increases RBC size and hemoglobin content. In the marrow, vadadustat increases release of reticulocytes compared with ESA administration. The red color intensity of erythroid cells indicates respective hemoglobin concentrations at each stage

As reported previously, vadadustat was noninferior to darbepoetin alfa with respect to hematological efficacy in the PRO_2_TECT and INNO_2_VATE phase 3 clinical trials.^35^, ^36^ The prespecified noninferiority safety outcomes in terms of MACE were met in the INNO_2_VATE trials but not in the PRO_2_TECT trials.^35^, ^36^ Time to CKD progression in the PRO_2_TECT trials was similar in the vadadustat‐ and darbepoetin‐treatment groups.^35^ Patients randomized to vadadustat had increased endogenous EPO, which through its ability to increase marrow erythroid progenitors, led to increased numbers of circulating RBCs. In addition to these EPO‐mediated effects, the present data provide evidence that vadadustat induced additional erythropoietic stimulation and other beneficial effects. Compared with patients randomized to darbepoetin, patients randomized to vadadustat had larger erythrocytes (MCV) that contained more hemoglobin (MCH), and also had increased numbers of reticulocytes (absolute reticulocyte count). These erythropoietic effects most likely result from the activity of vadadustat in the kidneys, liver, and bone marrow (Figure 3) in stimulating RBC production.

HIF‐2α regulates EPO gene transcription and subsequent EPO production.^39^, ^40^, ^41^ In ESA‐untreated patients with NDD‐CKD, vadadustat increased endogenous EPO production, with mean serum EPO concentrations between 16 and 21 mIU/mL, which is at, or slightly above, the upper limit of the range for healthy individuals (3.3–16.6 mIU/mL) and consistent with earlier results with vadadustat.^33^, ^34^ Vadadustat likely induces HIF‐2α in renal cortical myofibroblasts, which can produce EPO in response to decreased HIF‐PHD activity in diseased kidneys.^42^ Vadadustat can also induce EPO in the liver,^43^ most likely in hepatocytes, which are the main hepatic EPO‐producing cells.^44^

Patients randomized to vadadustat achieved and maintained target range hemoglobin concentrations during the primary and secondary evaluation periods in all four trials. The slightly above‐population reference range of serum EPO concentrations in both vadadustat‐treated and darbepoetin‐treated patients indicate that their EPO concentrations were sufficient for achieving the target hemoglobin range but not for achievement of a normal hemoglobin range. The patients' marrow erythroid cells likely had decreased EPO responsiveness compared with healthy individuals. Inflammatory cytokines can directly inhibit proliferation and survival of erythropoietic cells in the marrow,^10^, ^45^ and both vadadustat‐treated and darbepoetin‐treated groups had similar slight‐to‐moderate elevations of mean CRP concentrations during the trials, indicating that inflammation may have suppressed EPO responsiveness of the erythroid cells.

Nearly two‐thirds of total body reticulocytes are normally in the marrow,^46^ but phlebotomy‐induced blood loss physiologically results in early release of reticulocytes from the marrow into the blood that can be detected at 24 and 36 h after phlebotomy,^47^ well before EPO‐mediated expansion of erythroid progenitors can supply additional circulating reticulocytes. EPO may mediate some early reticulocyte release,^48^, ^49^ but consistently increased absolute reticulocytes in patients randomized to vadadustat compared with those randomized to darbepoetin indicate that another HIF‐mediated mechanism may be involved. Marrow reticulocytes pass through the endothelial cells of marrow venous sinuses to enter the blood,^50^ and early reticulocyte release is associated with changes in these marrow endothelial cells.^51^ HIFs with the potential to affect marrow endothelial cells are produced in the marrow by hematopoietic, stromal, and endothelial cells,^52^, ^53^, ^54^ and may be involved with vadadustat‐mediated increases in absolute reticulocyte counts.

In the present clinical trials with predetermined target hemoglobin concentrations and nonextensive blood loss, the increased reticulocytes in patients randomized to vadadustat represented 10%–20% of circulating reticulocytes and 0.3% of total circulating erythrocytes, thereby contributing only slightly to the increased mean MCV and MCH. MCVs of non‐reticulocytes in the RBC population of each study at Weeks 28 and 52 (Table S4) show the same MCV relationships as shown for the total RBC population (Table S3), further indicating that reticulocytes contributed very slightly to the MCV results. Compared with patients randomized to darbepoetin, those randomized to vadadustat produced uniformly larger erythrocytes (increased MCV with decreased RDW), with each cell containing more hemoglobin (increased MCH) and slightly fewer RBCs with relatively decreased RDWs despite achieving and maintaining similar hemoglobin concentrations.^35^, ^36^ Similar results for these erythrocyte indices were reported in other phase 3 trials comparing vadadustat with darbepoetin in patients with CKD.^55^, ^56^

These increases in erythrocyte size and hemoglobin content in the patients randomized to vadadustat compared with those randomized to darbepoetin are most consistent with improved iron delivery to erythroblasts, where increased intracellular iron enhances heme synthesis that, in turn, increases hemoglobin accumulation and cell size.^57^ Erythroblasts of vadadustat‐treated patients should contain increased heme due to HIF increasing transcription of the erythroid‐specific *ALAS‐2* gene^58^ and iron‐regulatory protein increasing translation of ALAS‐2 mRNAs.^59^ Increased erythroblast heme, in turn, induces enhanced heme‐regulated inhibitor‐controlled synthesis of globins and other proteins,^57^ leading to production of larger erythrocytes containing more hemoglobin (i.e., increased mean MCV and MCH) as observed in patients randomized to vadadustat compared with those randomized to darbepoetin.

Most iron supplied to erythroblasts is recycled from the degraded hemoglobin of senescent erythrocytes that have been phagocytosed by macrophages in a process that is not directly affected by HIF.^60^ The increased availability of iron with vadadustat versus darbepoetin was associated with increases in serum transferrin (measured as TIBC) and decreases in TSAT, with decreases in serum hepcidin and ferritin. Similar changes in these iron‐related proteins have been reported previously with other HIF‐PHD inhibitors as well as vadadustat.^55^, ^56^, ^61^, ^62^, ^63^ Hepcidin expression can be downregulated directly by HIF in iron deficiency,^64^ but hepcidin is indirectly downregulated by hypoxia^65^ during EPO‐mediated expansion of marrow erythroid cells^66^, ^67^ through their production of erythroferrone (ERFE), a hormone that suppresses liver hepcidin production.^68^ Hepcidin is also downregulated by a mechanism that does not involve ERFE when erythroid populations are not expanding^69^ and, in the case of vadadustat stimulation of erythropoiesis, ERFE is not required for hepcidin downregulation.^43^

ERFE from stimulated erythropoiesis may have contributed to decreased serum hepcidin in the first few months after initiating both vadadustat and darbepoetin treatments. At later times in the trials, however, persistent decreases in hepcidin for vadadustat‐treated and darbepoetin‐treated patients, and the relatively more prominent decreases in hepcidin with vadadustat treatment compared with darbepoetin treatment indicate a role for non–ERFE‐mediated effects. In this regard, vadadustat resulted in more pronounced increases in TIBC than darbepoetin at all times in each trial, suggesting that vadadustat induced the transferrin gene, a known transcriptional target of HIF‐1α,^70^ although other mechanisms may be involved with this increased TIBC.^43^ The decreased TSAT associated with increased transferrin, in turn, increases unsaturated and monosaturated transferrin, both of which decrease hepcidin production.^71^, ^72^, ^73^, ^74^ Thus, our data indicate that vadadustat increases total transferrin while decreasing its saturation with iron. This increase in desaturated transferrin decreases liver production of hepcidin which, in turn, allows improved mobilization of stored iron as shown by deceased serum ferritin.

Like most patients with CKD, participants in the INNO_2_VATE and PRO_2_TECT trials had elevated baseline serum ferritin and CRP concentrations due to inflammation and oral or intravenous iron supplementation. Furthermore, patients in the trials received oral and/or intravenous iron supplementation to maintain serum ferritin ≥100 ng/mL or TSAT ≥20%, as is common in the care of patients with CKD. Oral or intravenous iron use was similar in the two treatment groups throughout both studies. The serum CRP remaining stable in the 7–11 mg/L range in all four trials suggests that increasing serum ferritin at later times in both NDD‐CKD trials and the incident DD‐CKD trial was likely due to iron administrations rather than changes in inflammation. Because of this ongoing iron supplementation, the effects of vadadustat on oral iron absorption could not be determined. However, HIF‐2α plays key roles in duodenal iron absorption,^75^ and vadadustat upregulates HIF target genes encoding cytochrome B reductase, divalent metal transporter 1, and ferroportin in duodenal enterocytes.^43^ These changes combined with decreased hepcidin in vadadustat‐treated patients suggest that vadadustat likely increases iron absorption. Indeed, two other phase 3 trials demonstrated that vadadustat was noninferior to darbepoetin with respect to hematologic efficacy in patients with NDD‐CKD and DD‐CKD who had normal range serum ferritin concentrations.^55^, ^56^ However, clinical trials are needed to test whether vadadustat combined with oral iron supplementation can achieve and maintain target hemoglobin concentrations in CKD patients while reducing or eliminating intravenous iron administration.

In conclusion, results from four large phase 3 trials comparing the investigational drug vadadustat with an ESA, darbepoetin, demonstrated that vadadustat can increase endogenous EPO production, improve erythroblast iron availability, and increase circulating reticulocytes in patients with CKD and anemia. Although the underlying physiological mechanisms of these present results were not directly investigated, they are likely due to vadadustat effects on the kidneys, liver, and bone marrow (Figure 3).

## AUTHOR CONTRIBUTIONS

Contributions: Wolfgang C. Winkelmayer, Glenn M. Chertow, Kai‐Uwe Eckardt, Youssef M. K. Farag, and Patrick S. Parfrey conceived and designed the study. Wenli Luo acquired the data and Mark J. Koury, Wolfgang C. Winkelmayer, Glenn M. Chertow, Kai‐Uwe Eckardt, Todd Minga, and Wenli Luo analyzed the data. Mark J. Koury wrote the first draft and Mark J. Koury, Rajiv Agarwal, Glenn M. Chertow, Kai‐Uwe Eckardt, Steven Fishbane, Tomas Ganz, Volker H. Haase, Mark R. Hanudel, Patrick S. Parfrey, Pablo E. Pergola, Prabir Roy‐Chaudhury, James A. Tumlin, Robert Anders, Youssef M. K. Farag, Wenli Luo, Todd Minga, Christine Solinsky, Dennis L. Vargo, and Wolfgang C. Winkelmayer revised critically for important intellectual content. All authors contributed to the interpretation of the results. All authors provided final approval for the version to be published. The corresponding author attests to having full access to the study data and was responsible for the final decision to submit this manuscript for publication.

## CONFLICTS OF INTEREST

Steven Fishbane currently serves as a study consultant for Akebia Therapeutics Inc., AstraZeneca, and GlaxoSmithKline. Mark J. Koury, Rajiv Agarwal, Glenn M. Chertow, Kai‐Uwe Eckardt, Tomas Ganz, Volker H. Haase, Mark R. Hanudel, Patrick S. Parfrey, Prabir Roy‐Chaudhury, James A. Tumlin, and Wolfgang C. Winkelmayer currently serve as study consultants for Akebia Therapeutics Inc. Wenli Luo, Robert Anders, Christine Solinsky, and Dennis L. Vargo are employees of Akebia Therapeutics, Inc. Youssef M. K. Farag and Todd Minga were employees of Akebia Therapeutics, Inc., at the time the study was conducted.

## Supporting information


**Appendix S1** Supporting Information

## Data Availability

Proposals for access to original data should be sent to medicalinfo@akebia.com. Deidentified patient‐level data will be available 12 months after U.S. and E.U. approval to qualified researchers with an appropriate research proposal. The research proposal is subject to review by an independent review board with final approval by Akebia Therapeutics Inc.
