# Supplementary material for: Erythropoietic effects of vadadustat in patients with anemia associated with chronic kidney disease
Source: Am J Hematol. 2022 Jul 15;97(9):1178–88. doi: 10.1002/ajh.26644 (PMC9543410; doi:10.1002/ajh.26644)
Supplement: Supplementary file 1 — Appendix S1 Supporting Information [file AJH-97-1178-s001.docx]

**Supplementary Appendix**

**Table of Contents**

|  | Page no. (Suppl) |
| --- | --- |
| **Supplemental Tables** |  |
| Supplemental Table 1. Baseline characteristics of the trial participants in the PRO_2_TECT and INNO_2_VATE programs | 2 |
| Supplemental Table 2. Erythropoietin levels in darbepoetin alfa-treated patients at baseline and weeks 4, 12, 28, and 52 in the PRO_2_TECT and INNO_2_VATE programs | 3 |
| Supplemental Table 3. Erythrocyte parameters at baseline and weeks 28 and 52 in the PRO_2_TECT and INNO_2_VATE programs | 4 |
| Supplemental Table 4. Estimated non-reticulocyte MCVs (in fLs), assuming reticulocytes have mean MCV of 120 fL | 5 |
| Supplemental Table 5. Changes in iron-related outcomes in the PRO_2_TECT and INNO_2_VATE programs | 6, 7 |
| **Supplemental Figures** |  |
| Supplemental Figure 1. CONSORT flow chart for the ESA-untreated and ESA-treated PRO_2_TECT NDD-CKD trials. | 8 |
| Supplemental Figure 2. CONSORT flow chart for the incident and prevalent INNO_2_VATE DD-CKD trials. | 9 |
| Supplemental Figure 3. Bilirubin, lactate dehydrogenase, and C-reactive protein in the PRO_2_TECT and INNO_2_VATE programs | 10 |

**Supplemental Table 1**. Baseline characteristics of the trial participants in the PRO_2_TECT and INNO_2_VATE programs

|  | **PRO_2_TECT NDD-CKD** | | | | **INNO_2_VATE DD-CKD** | | | |
| --- | --- | --- | --- | --- | --- | --- | --- | --- |
| **Characteristic** | **ESA-untreated** | | **ESA-treated** | | **Incident dialysis** | | **Prevalent dialysis** | |
|  | **VADA** | **DA** | **VADA** | **DA** | **VADA** | **DA** | **VADA** | **DA** |
| Baseline, N | 879 | 872 | 862 | 863 | 181 | 188 | 1777 | 1777 |
| Age, years, mean (SD) | 65.2 (14.3) | 64.9  (13.7) | 67.3 (13.1) | 66.5 (13.5) | 56.5 (14.8) | 55.6 (14.6) | 57.9 (13.9) | 58.4 (13.8) |
| Male, n (%) | 404  (46.0) | 366  (42.0) | 394 (45.7) | 375 (43.5) | 107 (59.1) | 113 (60.1) | 990  (55.7) | 1004 (56.5) |
| Baseline hemoglobin, g/dL, mean (SD) | 9.1  (0.8) | 9.1  (0.8) | 10.4 (0.9) | 10.4  (0.9) | 9.4  (1.1) | 9.2  (1.1) | 10.3  (0.9) | 10.2 (0.8) |

CKD, chronic kidney disease; DA, darbepoetin alfa; DD, dialysis dependent; ESA, erythropoiesis-stimulating agent; NDD, non-dialysis dependent; SD, standard deviation; VADA, vadadustat.

**Supplemental Table 2.** Erythropoietin levels in darbepoetin alfa-treated patients at baseline and weeks 4, 12, 28, and 52 in the PRO_2_TECT and INNO_2_VATE programs

| **Erythropoietin** (mIU/mL) | **Baseline** | **Week 4** | **Week 12** | **Week 28** | **Week 52** |
| --- | --- | --- | --- | --- | --- |
| **ESA-untreated NDD-CKD trial** | | | | | |
| Observed, mean (SD) | 15.0 (14.0) | 17.8 (20.6) | 16.9 (24.4) | 20.3 (42.4) | 19.7 (35.6) |
| Change from baseline,  mean (SD) | NA | 3.0 (19.2) | 2.0 (21.5) | 6.0 (40.3) | 5.0 (35.2) |
| Change from baseline, 95% CI  *P* value | NA | (1.6, 4.3)  <.0001 | (0.5, 3.6)  0.0095 | (3.0, 9.0)  0.0001 | (1.9, 8.1)  0.0015 |
| **ESA-treated NDD-CKD trial** | | | | | |
| Observed, mean (SD) | 15.8 (19.9) | 16.2 (14.8) | 16.9 (49.3) | 17.0 (25.8) | 17.6 (27.1) |
| Change from baseline,  mean (SD) | NA | 0.4 (15.8) | 1.1 (50.4) | 1.7 (28.2) | 1.2 (27.6) |
| Change from baseline, 95% CI  *P* value | NA | (-0.7, 1.5)  0.4976 | (-2.5, 4.7)  0.5334 | (-0.3, 3.8)  0.1011 | (-1.2, 3.6)  0.3211 |
| **Incident DD-CKD trial** | | | | | |
| Observed, mean (SD) | 21.1 (48.4) | 28.4 (53.3) | 21.4 (43.6) | 24.0 (78.9) | 26.7 (61.4) |
| Change from baseline,  mean (SD) | NA | 7.5 (70.4) | 1.4 (64.7) | 4.5 (46.5) | 10.9 (66.2) |
| Change from baseline, 95% CI  *P* value | NA | (-3.1, 18.1)  0.1654 | (-8.5, 11.2)  0.7823 | (-2.9, 11.9)  0.2304 | (-2.8, 24.6)  0.1169 |
| **Prevalent DD-CKD trial** | | | | | |
| Observed, mean (SD) | 26.4 (77.0) | 26.6 (89.3) | 23.7 (64.1) | 28.3 (122.0) | 24.1 (64.0) |
| Change from baseline,  mean (SD) | NA | 0.7 (100.5) | -2.5 (93.7) | 3.3 (137.7) | -0.8 (89.8) |
| Change from baseline, 95% CI  *P* value | NA | (-4.1, 5.6)  0.7682 | (-7.1, 2.1)  0.2780 | (-3.7, 10.3)  0.3574 | (-5.8, 4.3)  0.7637 |

CI, confidence interval; CKD, chronic kidney disease; DD, dialysis dependent; ESA, erythropoiesis-stimulating agent; NA, not applicable; NDD, non-dialysis dependent; SD, standard deviation.

**Supplemental Table 3.** Erythrocyte parameters at baseline and weeks 28 and 52 in the PRO_2_TECT and INNO_2_VATE programs

| **Parameter,**  **mean (SD)** | **Baseline** | | **Week 28** | | **Week 52** | |
| --- | --- | --- | --- | --- | --- | --- |
|  | **VADA** | **DA** | **VADA** | **DA** | **VADA** | **DA** |
| **ESA-untreated NDD-CKD trial** | | | | | | |
| Hemoglobin (g/dL) | 9.1 (0.8) | 9.1 (0.8) | 10.5 (1.1) | 10.4 (1.2) | 10.6 (1.1) | 10.6 (1.2) |
| RBCs (× 10^6^/µL) | 3.1 (0.4) | 3.1 (0.4) | 3.5 (0.5) | 3.5 (0.5) | 3.5 (0.5) | 3.6 (0.5) |
| Reticulocytes  (× 10^3^/µL) | 52.2 (25.5) | 52.4 (24.8) | 57.3 (26.7) | 48.6 (27.8) | 55.5 (25.5) | 49.2 (28.8) |
| MCV (fL) | 92.2 (7.3) | 91.6 (7.2) | 93.3 (7.5) | 92.0 (7.2) | 94.0 (7.5) | 92.5 (7.3) |
| MCH (pg/cell) | 30.1 (3.4) | 29.9 (2.6) | 30.2 (2.6) | 29.7 (2.57) | 30.4 (2.7) | 29.8 (2.7) |
| RDW (%) | 15.5 (2.2) | 15.6 (2.2) | 15.6 (2.0) | 16.1 (2.2) | 15.6 (1.9) | 16.1 (2.8) |
| **ESA-treated NDD-CKD trial** | | | | | | |
| Hemoglobin (g/dL) | 10.4 (0.9) | 10.4 (0.9) | 10.9 (1.1) | 10.8 (1.1) | 10.8 (1.1) | 10.8 (1.2) |
| RBCs (× 10^6^/µL) | 3.5 (0.5) | 3.5 (0.4) | 3.6 (0.5) | 3.6 (0.5) | 3.6 (0.5) | 3.6 (0.5) |
| Reticulocytes  (× 10^3^/µL) | 52.7 (27.6) | 52.3 (31.3) | 58.0 (30.2) | 48.9 (29.5) | 55.5 (28.2) | 48.5 (31.7) |
| MCV (fL) | 92.5 (7.3) | 93.0 (6.7) | 94.2 (7.4) | 93.2 (6.7) | 94.4 (7.9) | 93.1 (7.0) |
| MCH (pg/cell) | 30.0 (2.6) | 30.2 (2.4) | 30.6 (2.6) | 30.2 (2.4) | 30.6 (2.6) | 30.2 (2.6) |
| RDW (%) | 15.8 (2.0) | 15.7 (2.0) | 15.5 (1.9) | 15.8 (2.1) | 15.52 (1.9) | 15.85 (2.1) |
| **Incident DD-CKD trial** | | | | | | |
| Hemoglobin (g/dL) | 9.4 (1.1) | 9.2 (1.1) | 10.3 (1.3) | 10.7 (1.2) | 10.4 (1.3) | 10.6 (1.2) |
| RBCs (× 10^6^/µL) | 3.1 (0.4) | 3.1 (0.5) | 3.3 (0.5) | 3.5 (0.5) | 3.4 (0.5) | 3.5 (0.5) |
| Reticulocytes  (× 10^3^/µL) | 51.4 (26.3) | 51.8 (36.5) | 59.7 (35.6) | 49.9 (27.0) | 55.4 (31.9) | 52.5 (29.9) |
| MCV (fL) | 91.6 (5.5) | 91.7 (5.7) | 95.0 (6.4) | 94.0 (6.7) | 94.5 (6.8) | 94.9 (6.8) |
| MCH (pg/cell) | 30.2 (2.0) | 30.2 (2.1) | 31.1 (2.1) | 30.6 (2.4) | 31.1 (2.5) | 30.8 (2.6) |
| RDW (%) | 15.7 (1.9) | 15.6 (1.9) | 15.2 (1.6) | 16.0 (1.8) | 15.1 (1.4) | 16.0 (2.0) |
| **Prevalent DD-CKD trial** | | | | | | |
| Hemoglobin (g/dL) | 10.3 (0.9) | 10.2 (0.8) | 10.4 (1.2) | 10.6 (1.1) | 10.5 (1.2) | 10.6 (1.2) |
| RBCs (× 10^6^/µL) | 3.3 (0.4) | 3.3 (0.4) | 3.3 (0.4) | 3.4 (0.4) | 3.4 (0.5) | 3.4 (0.5) |
| Reticulocytes  (× 10^3^/µL) | 55.5 (29.9) | 55.2 (28.7) | 55.7 (26.6) | 51.2 (28.6) | 54.7 (28.2) | 47.0 (26.5) |
| MCV (fL) | 94.8 (6.9) | 94.8 (6.3) | 96.1 (7.1) | 95.4 (6.7) | 96.1 (7.2) | 95.6 (6.8) |
| MCH (pg/cell) | 31.1 (2.4) | 31.1 (2.3) | 31.5 (2.5) | 31.2 (2.4) | 31.5 (2.6) | 31.2 (2.5) |
| RDW (%) | 15.8 (1.9) | 15.8 (1.8) | 15.4 (1.8) | 16.3 (1.9) | 15.6 (1.9) | 16.4 (1.9) |

CKD, chronic kidney disease; DA, darbepoetin alfa; DD, dialysis dependent; ESA, erythropoiesis-stimulating agent; MCH, mean corpuscular hemoglobin; MCV, mean corpuscular volume; NDD, non-dialysis dependent; RBC, red blood cell; RDW, red cell distribution width; SD, standard deviation; VADA, vadadustat.

**Supplemental Table 4.** Estimated non-reticulocyte MCVs (in fLs), assuming reticulocytes have mean MCV of 120 fL

| Trial/group | Baseline | | Week 28 | | Week 52 | |
| --- | --- | --- | --- | --- | --- | --- |
|  | All RBCs | Non-reticulocytes | All RBCs | Non-reticulocytes | All RBCs | Non-reticulocytes |
| **ESA-untreated NDD-CKD trial** | | | | | | |
| Vadadustat | 92.2 | 91.7 | 93.3 | 92.8 | 94.0 | 93.6 |
| Darbepoetin Alfa | 91.6 | 91.1 | 92.0 | 91.5 | 92.5 | 92.0 |
| **ESA-treated NDD-CKD trial** | | | | | | |
| Vadadustat | 92.5 | 92.0 | 94.2 | 93.8 | 94.4 | 94.0 |
| Darbepoetin Alfa | 93.0 | 92.5 | 93.2 | 92.7 | 93.1 | 92.6 |
| **Incident DD-CKD trial** | | | | | | |
| Vadadustat | 91.6 | 91.1 | 95.0 | 94.6 | 94.5 | 94.1 |
| Darbepoetin Alfa | 91.7 | 91.2 | 94.0 | 93.6 | 94.9 | 94.5 |
| **Prevalent DD-CKD trial** | | | | | | |
| Vadadustat | 94.8 | 94.4 | 96.1 | 95.7 | 96.1 | 95.7 |
| Darbepoetin Alfa | 94.8 | 94.4 | 95.4 | 95.0 | 95.6 | 95.2 |

CKD, chronic kidney disease; DD, dialysis dependent; ESA, erythropoiesis-stimulating agent; MCV, mean corpuscular volume; NDD, non-dialysis dependent; RBC, red blood cell.

**Supplemental Table 5**. Changes in iron-related outcomes in the PRO_2_TECT and INNO_2_VATE programs

| **Parameter** | **Baseline** | | **Primary evaluation period (weeks 24-36)** | | | | **Secondary evaluation period (weeks 40-52)** | | | |
| --- | --- | --- | --- | --- | --- | --- | --- | --- | --- | --- |
|  | **VADA,**  **mean**  **(SD)** | **DA,**  **mean**  **(SD)** | **VADA,**  **LS mean (SEM)** | **DA,**  **LS mean (SEM)** | **Difference (VADA–DA)**  **LS mean**  **(95% CI)** | ***P* value** | **VADA,**  **LS mean (SEM)** | **DA,**  **LS mean (SEM)** | **Difference (VADA–DA)**  **LS mean**  **(95% CI)** | ***P* value** |
| **ESA-untreated NDD-CKD trial** | | | | | | | | | | |
| TIBC, μg/dL | 240.4 (48.2) | 241.1 (43.8) | n = 731  34.6 (1.8) | n = 746  2.7 (1.8) | 31.9  (28.4, 35.4) | < .0001 | n = 617  34.1 (2.1) | n = 628  3.6 (2.0) | 30.5  (26.4, 34.6) | < .0001 |
| Hepcidin, ng/mL | 105.5 (100.3) | 104.6 (100.9) | n = 687  –39.8 (3.6) | n = 703  –13.8 (3.6) | –26.1  (–33.2,  –18.9) | < .0001 | n = 464  –41.5 (4.4) | n = 447  –20.9 (4.5) | –20.6  (–29.4,  –11.8) | < .0001 |
| Ferritin, ng/mL | 368.1 (293.7) | 360.3 (286.9) | n = 732  –76.6 (12.1) | n = 748  –46.9 (12.1) | –29.7  (–53.6, –5.7) | .01 | n = 620  –83.8 (14.2) | n = 628  –41.6 (14.2) | –42.2  (–70.5,  –13.9) | .003 |
| Serum iron, μg/dL | 73.8  (28.9) | 73.6  (24.4) | n = 731  1.9 (1.3) | n = 747  –0.7 (1.3) | 2.7  (0.0, 5.3) | .05 | n = 621  0.3 (1.5) | n = 629  0.0 (1.5) | 0.3  (–2.8, 3.4) | .85 |
| TSAT, % | 30.9  (9.6) | 31.0  (10.0) | n = 731  –3.0 (0.5) | n = 746  –0.3 (0.5) | –2.8  (–3.8, –1.7) | < .0001 | n = 617  –3.4 (0.6) | n = 627  0.1 (0.6) | –3.5  (–4.6, –2.3) | < .0001 |
| **ESA-treated NDD-CKD trial** | | | | | | | | | | |
| TIBC, μg/dL | 242.2 (44.1) | 224.8 (42.5) | n = 761  34.3 (1.5) | n = 782  0.2 (1.5) | 34.1  (30.8, 37.4) | < .0001 | n = 640  30.0 (1.8) | n = 655  –0.1 (1.8) | 30.1  (26.3, 33.9) | < .0001 |
| Hepcidin, ng/mL | 100.4 (90.2) | 106.2 (99.98) | n = 734  –37.3 (3.2) | n = 749  –8.9 (3.1) | –28.5  (–35.3,  –21.6) | < .0001 | n = 436  –38.7 (3.8) | n = 442  –20.2 (3.9) | –18.5  (–26.8,  –10.2) | < .0001 |
| Ferritin, ng/mL | 368.5 (285.3) | 383.2 (319.4) | n = 762  –75.3 (9.9) | n = 786  –40.5 (9.8) | –34.8  (–56.2,  –13.5) | .001 | n = 642  –39.6 (12.1) | n = 657  –38.4 (11.9) | –1.2  (–27.0, 24.6) | .93 |
| Serum iron, μg/dL | 78.6  (25.3) | 79.0  (27.1) | n = 762  –0.4 (1.1) | n = 784  –3.3 (1.1) | 2.87  (0.40, 5.35) | .02 | n = 642  1.0 (1.4) | n = 657  –3.4 (1.3) | 4.5  (1.6, 7.4) | .002 |
| TSAT, % | 32.8  (9.7) | 32.9  (10.5) | n = 761  –3.7 (0.5) | n = 782  –0.6 (0.4) | –3.1  (–4.1, –2.1) | < .0001 | n = 640  –3.0 (0.5) | n = 655  –0.9 (0.5) | –2.1  (–3.3, –0.9) | .0006 |
| **Incident DD-CKD trial** | | | | | | | | | | |
| TIBC, μg/dL | 227.3 (39.5) | 226.5 (41.2) | n = 152  24.1 (3.9) | n = 167  –1.7 (3.8) | 25.8  (18.2, 33.5) | < .0001 | n = 131  19.2 (4.5) | n = 142  –4.4 (4.3) | 23.6  (14.9, 32.3) | < .0001 |
| Hepcidin, ng/mL | 122.4 (109.5) | 126.9 (111.2) | n = 150  –17.8 (9.2) | n = 160  –6.2 (9.1) | –11.6  (–29.7, 6.5) | .21 | n = 123  –22.4 (9.9) | n = 129  –16.8 (9.9) | –5.6  (–24.6, 13.4) | .56 |
| Ferritin, ng/mL | 469.7 (316.9) | 527.8 (401.1) | n = 152  42.3 (34.3) | n = 167  80.0 (33.3) | –37.7  (–104.8, 29.4) | .27 | n = 131  79.4 (44.1) | n = 142  92.6 (42.8) | –13.2  (–99.9, 73.5) | .77 |
| Serum iron, μg/dL | 71.2 (25.3) | 77.6 (33.5) | n = 152  3.5 (3.3) | n = 167  5.3 (3.2) | –1.8  (–8.4, 4.8) | .59 | n = 131  8.2 (3.5) | n = 142  6.0 (3.3) | 2.2  (–4.6, 9.0) | .52 |
| TSAT, % | 31.3 (9.4) | 34.2 (12.7) | n = 152  –0.7 (1.3) | n = 167  3.9 (1.3) | –4.6  (–7.2, –2.0) | < .001 | n = 131  1.6 (1.4) | n = 142  4.0 (1.4) | –2.3  (–5.2, 0.5) | .11 |
| **Prevalent DD-CKD trial** | | | | | | | | | | |
| TIBC, μg/dL | 210.1 (36.1) | 211.6 (36.4) | n = 1535  30.7 (1.1) | n = 1587  1.7 (1.1) | 29.0  (26.8, 31.2) | < .0001 | n = 1411  28.2 (1.2) | n = 1481  1.4 (1.2) | 26.8  (24.4, 29.2) | < .0001 |
| Hepcidin, ng/mL | 193.9 (140.1) | 190.4 (135.9) | n = 1496  –53.5 (3.8) | n = 1512  –33.7 (3.8) | –19.8  (–27.6, –12.0) | < .0001 | n = 1224  –66.2 (4.2) | n = 1260  –43.6 (4.2) | –22.6  (–30.9, –14.2) | < .0001 |
| Ferritin, ng/mL | 846.8 (562.6) | 840.7 (538.5) | n = 1539  –79.4 (13.8) | n = 1590  –39.9 (13.7) | –39.5  (–67.1, –11.9 | .005 | n = 1414  –102.5 (13.9) | n = 1487  –57.8 (13.7) | –44.7  (–72.2, –17.2) | .002 |
| Serum iron, μg/dL | 79.6 (29.3) | 79.0 (28.2) | n = 1538  –0.4 (0.9) | n = 1589  –6.0 (0.9) | 5.7  (3.8, 7.6) | < .0001 | n = 1413  –3.1 (1.0) | n = 1486  –6.2 (1.0) | 3.1  (1.2, 5.1) | .002 |
| TSAT, % | 38.1 (13.4) | 37.6 (13.2) | n = 1535  –4.5 (0.4) | n = 1587  –2.7 (0.4) | –1.7  (–2.6, –0.9) | < .0001 | n = 1411  –5.4 (0.5) | n = 1481  –2.8 (0.5) | –2.6  (–3.5, –1.7) | < .0001 |

DA, darbepoetin alfa; DD-CKD, dialysis-dependent chronic kidney disease; ESA, erythropoiesis-stimulating agent; LS, least squares NDD-CKD, non–dialysis-dependent chronic kidney disease; TIBC, total iron-binding capacity; TSAT, transferrin saturation; VADA, vadadustat.

**Supplemental Figure 1. CONSORT flow chart for the ESA-untreated and ESA-treated PRO_2_TECT NDD-CKD trials.**

*ESA-untreated NDD-CKD trial panel*

**
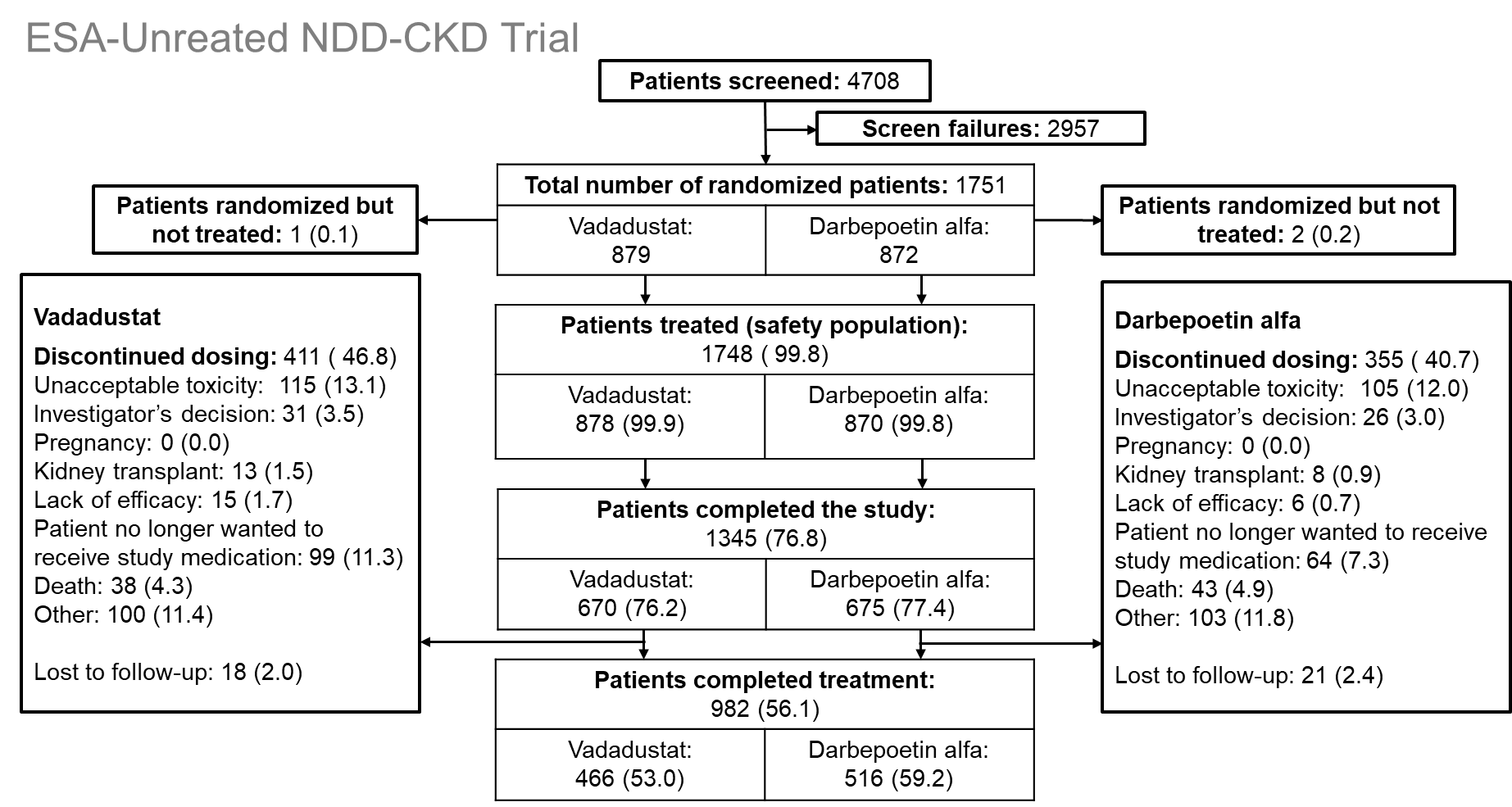
**

*ESA-treated NDD-CKD trial panel*

**
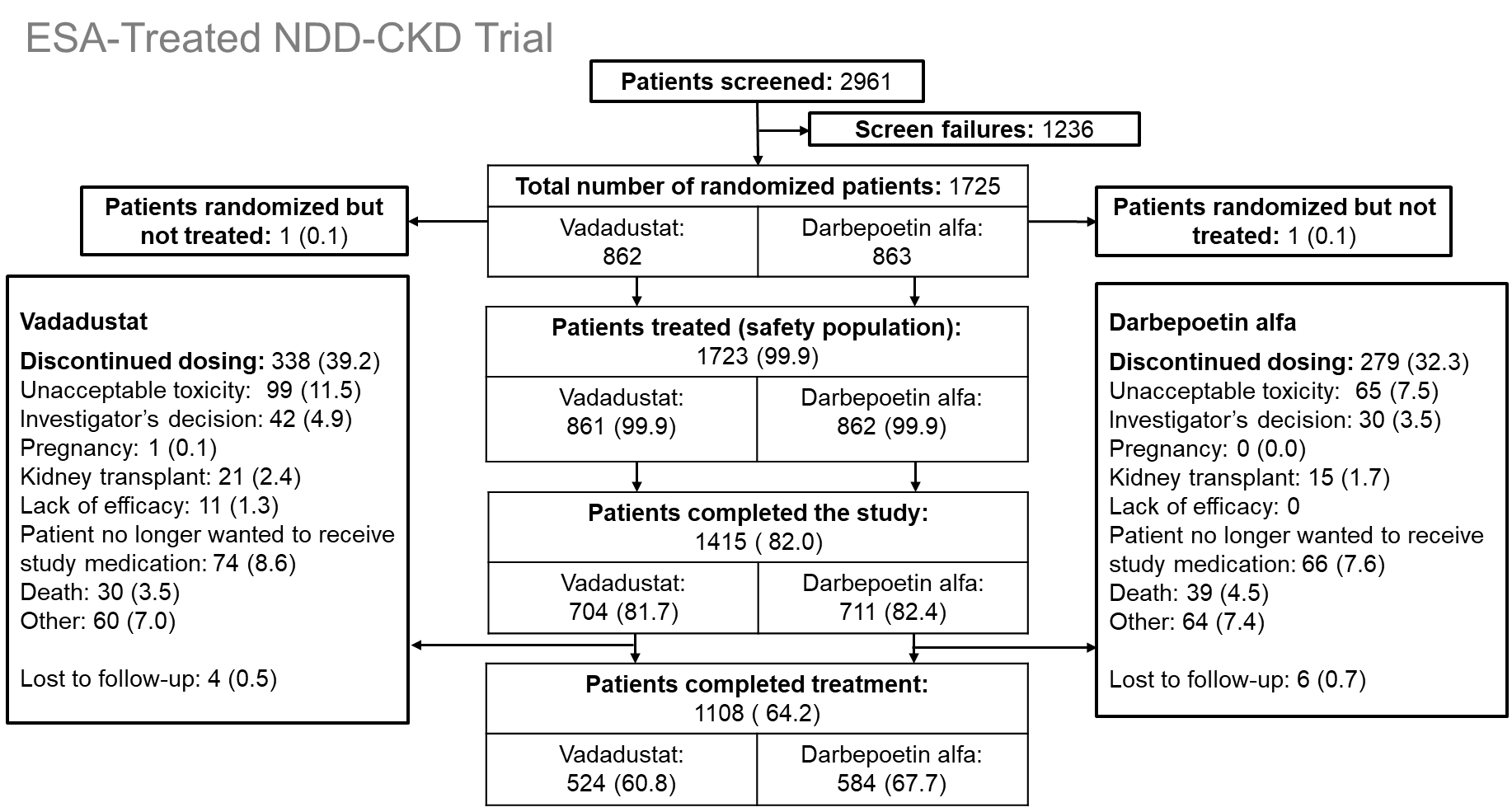
**

Note: Numbers in parentheses represent percentage of total randomized patients in either vadadustat or darbepoetin alfa groups.

ESA, erythropoiesis-stimulating agent; NDD-CKD, non–dialysis-dependent chronic kidney disease.

**Supplemental Figure 2. CONSORT flow chart for the incident and prevalent INNO_2_VATE DD-CKD trials.**

*Incident DD-CKD trial panel*

*Prevalent DD-CKD trial panel*

Note: Numbers in parentheses represent percentage of total randomized patients in either vadadustat or darbepoetin alfa groups.

DD-CKD, dialysis-dependent chronic kidney disease.

**Supplemental Figure 3.** **Bilirubin, lactate dehydrogenase, and C-reactive protein in the PRO_2_TECT and INNO_2_VATE programs**

A, ESA-untreated NDD-CKD trial; B, ESA-treated NDD-CKD trial; Incident DD-CKD trial; Prevalent DD-CKD trial.

Data points represent means, and error bars represent standard error of the mean.

DA, darbepoetin alfa; DD-CKD, dialysis-dependent chronic kidney disease; ESA, erythropoiesis-stimulating agent; NDD-CKD, non–dialysis-dependent chronic kidney disease.
